# Genetic differentiation between cave and surface-dwelling populations of *Garra barreimiae *(Cyprinidae) in Oman

**DOI:** 10.1186/1471-2148-11-172

**Published:** 2011-06-20

**Authors:** Luise Kruckenhauser, Elisabeth Haring, Robert Seemann, Helmut Sattmann

**Affiliations:** 1 1st Zoological Department, Laboratory of Molecular Systematics, Museum of Natural History Vienna, Burgring 7, 1010 Vienna, Austria; 2 Department of Mineralogy and Petrography, Museum of Natural History Vienna, Burgring 7, 1010 Vienna, Austria; 3 3rd Zoological Department, Museum of Natural History Vienna, Burgring 7, 1010 Vienna, Austria

## Abstract

**Background:**

Phenotypic similarities among cave-dwelling animals displaying troglomorphic characters (e.g. reduced eyes and lack of pigmentation) have induced a long-term discussion about the forces driving convergent evolution. Here we introduce *Garra barreimiae *Fowler & Steinitz, 1956, as an interesting system to study the evolution of troglomorphic characters. The only hitherto known troglomorphic population of this species lives in Al Hoota Cave (Sultanate of Oman) close to a surface population. As a first approach, we assessed the genetic differentiation between the two morphotypes of *G. barreimiae *to determine whether gene flow still occurs.

**Results:**

We analysed the mitochondrial control region (CR). In *G. barreimiae *the CR starts immediately downstream of the *tRNA-Thr *gene, while the *tRNA-Pro *gene is missing at this genomic location. Interestingly, a putative *tRNA-Pro *sequence is found within the CR. The phylogenetic analyses of the CR sequences yielded a tree divided into three clades: Clade 1 has a high genetic distance to the other clades and contains the individuals of three populations which are separated by a watershed from all the others. Clade 2 comprises the individuals from Wadi Bani Khalid, the geographically most remote population. Clade 3 comprises all other populations investigated including that of Al Hoota Cave. The latter forms a haplogroup which also includes individuals from the adjacent surface population.

**Conclusions:**

Our data indicates that the troglomorphic cave population is of quite recent origin supporting the hypothesis that selection drives the fast evolution of troglomorphic traits. In this context pleiotropic effects might play an important role as it has been shown for *Astyanax*. There seems to be some gene flow from the cave population into the adjacent surface populations. One blind individual, found at a surface locality geographically distinct from Al Hoota Cave, is genetically differentiated from the other blind specimens indicating the probable existence of another cave population of *G. barreimiae*. The phylogeographic analyses show that while some of the surface populations are either still in contact or have been until recently, the population Wadi Bani Khalid is genetically separated. One group consisting of three populations is genetically highly differentiated questioning the conspecifity with *G*. *barreimiae*.

## Background

Troglomorphic forms of animals have fascinated humans ever since the first cave-dwelling animals were discovered [1,2]. The most striking features of many troglobionts are the reduction of eyes and of surficial pigmentation [3]. The discussion about the forces driving convergent evolution of reduced eyes and loss of pigmentation in typical cave-living animals was initiated very early in the history of evolutionary theory [4]. In general, two competing hypotheses have been proposed [5]: i) The lack of selective constraints leads to degeneration due to accumulation of mutations which are fixed by genetic drift. ii) The troglomorphic traits provide selective advantages. The latter explanation includes speculations on how reduced eyes would be advantageous, e.g., no infectious diseases of the eyes or energetic economy [6]. Pleiotropic effects have also been discussed, i.e. that the same genes are involved in eye development and in the formation of other constructive traits (e.g., taste bud and forebrain development [7-10]).

To date about 86 troglomorphic fish species from 18 different families have been described [1], displaying various degrees of troglomorphism. There are, however, also some cave-living fishes without troglomorphic phenotype [11]. Most studies conducted on cave fishes were carried out on species of the genus *Astyanax*, which became model organisms for investigating many different aspects of the development of troglomorphic characters (for a review see [5]). The data suggest that eye loss in *Astyanax *is mainly driven by natural selection; the loss of pigmentation seems more likely to be due to the accumulation of neutral mutations [5]. Nevertheless, convergent evolution of various troglomorphic species might be driven by different evolutionary mechanisms. Thus, investigating other species groups is important.

Here, we introduce another interesting system to study the evolution of troglomorphic populations. *Garra barreimiae *Fowler & Steinitz, 1956 is a fish species endemic in the southeastern Arabian Peninsula; it is listed as vulnerable by the IUCN 2010 (IUCN Red List of Threatened Species. Version 2010.4.). Surface populations of this species with well developed eyes and proper pigmentation are common in ponds and rivulets of the Hajar Mountains [12,13]. In 1980, however, A. Dunsire and M. Gallagher discovered one hypogean, troglomorphic population [14]. These eyeless and unpigmented fish were assigned to the same species as the surface populations, because besides the troglomorphic traits no further striking morphological differences were detected [14]. The juveniles of the eyeless form display eyes which become reduced during the growth to the adult stage [14,15]. Hence, the adults have no externally visible eyes, and the optic lobes are not fully developed [14]. Furthermore they have a weak squamation [9], a trait that is defined by a reduced number and size of scales [16].

**Figure 1:**
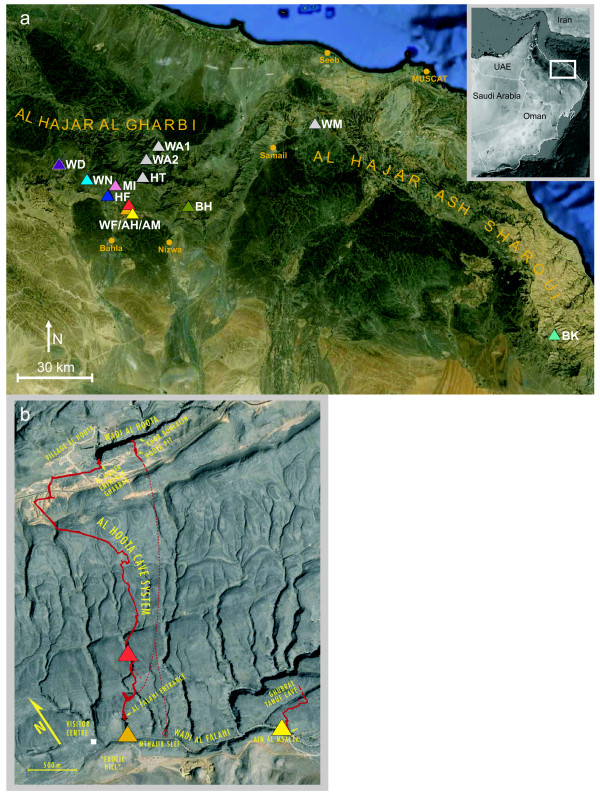
**Sampling sites in the Sultanate of Oman (a) and a detailed map of the Al Hoota Cave system and the adjacent areas (b).** Wadi Mansah, WM; Wadi Hat, HT; Wadi Bani Awf1, WA1; Wadi Bani Awf2, WA2; Wadi Bani Khalid, BK; Al Hoota Cave, AH; Wadi Al Falahi, WF; Ain Al Msalla, AM; Wadi An Nakhar, WN; Wadi Bani Habib, BH; Hamra Al Falaj, HF; Wadi Misfat, MI; Wadi Dhum, WD. The colour codes are the same as in Figure 4. The white rectangular in the insert shows the location of the area in a map of the Arabian Peninsula. In the detailed map the sampling sites Al Hoota Cave (red), Wadi Al Fallahi (orange) and Ain Al Msalla (yellow) are depicted.

The geographic coordinates of Dunsire's finding (23 05 N, 57 21 E) coincide with the Al Hoota Cave System near Al Hamra. Thus, this locality represents the only hitherto known troglomorphic population of *G. barreimiae *[12,13]. Al Hoota Cave System has connected subterraneous lakes and is situated in the Ad Dakhliyyah Region in the Western Al Hajar Mountains (Al Hajar Al Gharbi) northwest of the town Nizwa and east of Al Hamra (Figure 1). The Al Hoota population of blind fish lives very close to a surface population in the same natural drainage system. It is unknown to what degree the development of functional eyes in *G. barreimiae *is genetically determined or whether it might be induced by environmental factors. An indication of a potential influence of light was reported by [14]: Individuals originating from the cave population, which were raised in aquaria and exposed to light, showed a somewhat increased level of melanin production and development of the optic lobes (compared to individuals from the cave dissected immediately after capture).

When trying to understand the evolution of troglomorphic forms it is crucial to consider the time frames in which these populations separate from surface populations. In some of the populations of *Astyanax *and *Sinocyclocheilus *the split between the troglomorphic and the adjacent surface populations occurred quite recently [17-19], while in others the lineages apparently diverged long ago [18,19].

As a first approach we want to assess the genetic differentiation between the two morphotypes of *G. barreimiae *and determine whether gene flow presently occurs. Lack of gene flow would favour the hypothesis that the developmental differences are mainly genetically determined. The aim of the study was to analyse the phylogenetic relationships of cave and surface fish populations, particularly from Al Hoota Cave and from adjacent areas. Furthermore, the comprehensive phylogeographic analysis of the species required inclusion more distant localities in the Sultanate of Oman (Figure 1).

## Results

### Sequence analysis of the control region (CR)

To determine the part of the CR with the highest amount of variability we analysed a 1364 bp section of the mitochondrial DNA including the whole CR from five individuals. This analysis revealed an unexpected gene order upstream of the CR. In most fish species analysed so far the tRNA genes for Threonine and Proline (*tRNA-Thr *and *tRNA-Pro*) are located between the *cytochrome b *gene and the CR. Thus, both genes usually provide good primer binding sites for the amplification of the CR (in combination with primers binding downstream of the CR, in the *tRNA-Phe *or the *12rRNA *gene). In *G. barreimiae*, however, the CR starts immediately after the *tRNA-Thr*, while *tRNA-Pro *is missing at this genomic location. Interestingly, a putative *tRNA-Pro *(72 bp) sequence is found within the CR, 254 bp upstream of the 3'-end. This sequence contains the anticodon (tgg) and complementary sections stabilizing the cloverleaf structure as expected for a functional *tRNA-Pro*. The putative *tRNA-Pro *gene has an antiparallel orientation as has been described for other vertebrates and the sequence identity to the *tRNA-Pro *gene of *Cyprinus carpio *[20] is about 80%. Exchange of the positions of tRNA genes has been reported for many animal species (e.g., [21-23] and references therein) and seems to be the most common form of rearrangements of mitochondrial genomes. In fish the first report of tRNA rearrangements was for *Gonostoma gracile *(Teleostei: Stomiiformes; [24]) followed by similar observations in numerous fish species (e.g., [25] and references therein). To date, however, translocation of a tRNA gene into the non-coding CR has not been reported.

Due to the low quality of the DNA from some specimens we had to use a rather short fragment to obtain the DNA sequence of as many individuals as possible. The comparison of the five complete CR sequences showed that the variation in the 5'-section was similar to that in the 3'-section. For the 5'-part of the CR the primer binding was more efficient, and this fragment (CR1: 440 bp) was therefore chosen for the population analyses (length of the alignment 400 bp). DNA was extracted from 213 specimens of the genus *Garra *(including five *G. rufa *as outgroup)and the CR1 was successfully amplified from 150 individuals (145 *G. barreimiae*, five *G. rufa*). From each of the resulting haplogroups we chose several specimens to sequence an additional overlapping 545 bp fragment (CR2) to obtain information from a longer sequence (CR1+CR2; length of alignment 861 bp). The sequence of CR1 and CR2 was determined from a total of 33 individuals (32 *G. barreimiae*, one *G*. *rufa*).

### Phylogenetic analyses

**Figure 2:**
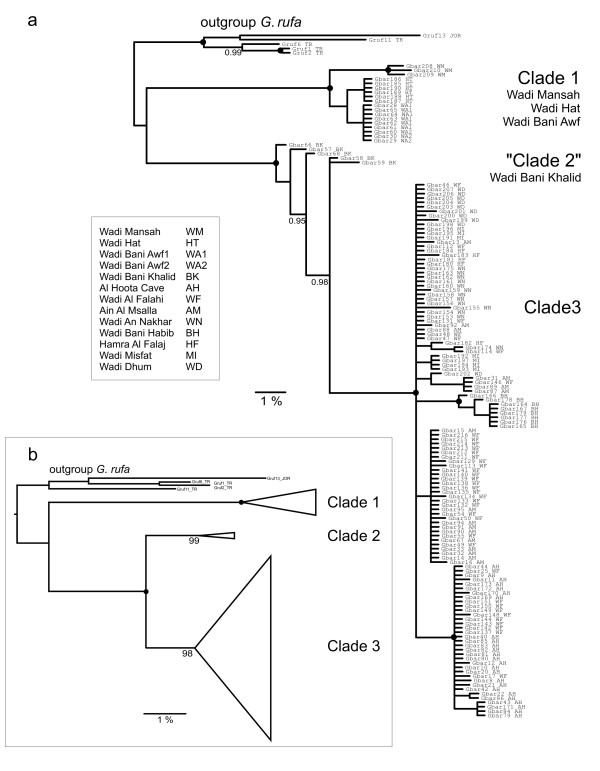
**Bayesian tree (a) and NJ tree (b) based on CR1 sequences**. Posterior probabilities of the main clades above 0.95 (a) and bootstrap values in % (b) are indicated at the nodes. Black dots at the nodes indicate posterior probabilities of 1.00 or bootstrap values of 100%, respectively. Designation of individuals includes individual number and locality abbreviations.

The analyses of the CR1 region resulted in the BI and NJ trees shown in Figure 2a and 2b. The two trees do not differ in their topology with one exception: One clade in the NJ tree (supported by high bootstrap values) decays in the BI analyses and appears in a ladderized manner. This may be an artefact of the BI analysis due to the short length of the sequences. The other two main clades are highly supported in both trees. In the following we refer to these three groups as clades. Clade 1 comprises three subclades, (1) nine individuals from Wadi Bani Awf (two distinct localities), (2) six individuals from Wadi Hat which is located in Al Hajar Al Gharbi, and (3) three individuals from Wadi Mansah located in the region between Al Hajar Al Gharbi and Al Hajar Ash Sharqi (Figure 1a). The latter subclade is the sister group of the former two. All individuals from Wadi Bani Awf share the same haplotype, which is very similar to the single haplotype of the Wadi Hat individuals. All populations of clade 1 are located at the northern side of the Al Jabal al Akhdar mountain range. Clade 2 comprises the individuals from Wadi Bani Khalid, the geographically most remote population about 200 km to the east of Al Hoota area. Clade 3 comprises all other localities from the southern side of Al Hajar Al Gharbi. The internal structure of clade 3 is rather bush-like. Only two highly supported haplogroups are found: One comprises all individuals from Wadi Bani Habib (located in the Al Jabal Al Akhdar), the other one all individuals from Al Hoota Cave and ten from the adjacent Wadi Al Falahi. The remaining haplotypes from Wadi Al Falahi are also included in clade 3.

**Figure 3:**
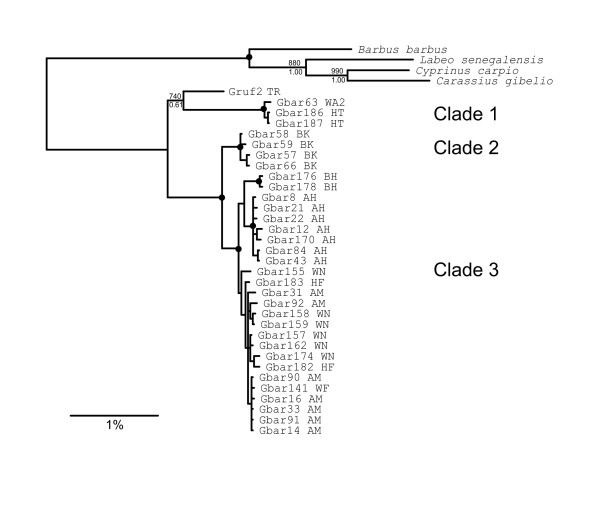
**Bayesian tree of the CR1+CR2 sequences**. Posterior probabilities as well as bootstrap values of a NJ tree based on this data set are indicated for the main clades. Black dots at the nodes indicate posterior probabilities of 1.00 and bootstrap values of 1000. Designation of individuals includes individual number and locality abbreviations.

The analyses of the CR1+CR2 data set were performed to obtain a better resolution of the relationships between the three major lineages. They are based on 32 *G. barreimiae *individuals representing the three major clades (Figure 3). In the resulting BI tree all three clades are highly supported. Also the sister group relationship of clades 2 and 3 obtained strong support. There is no statistical support for clade 1 grouping together with the two other *G. barreimiae *clades. Moreover, the distances (average p-distances) between clade 1 and the other two *G. barreimiae *clades are even higher (11% in both cases) than those between *G*. *rufa *and clade 1 (9%). The distances of clade 2 and 3 to *G. rufa *are smaller (9%). Therefore, more distantly related outgroup species (*Cyprinus carpio*, *Carassius gibelio*, *Labeo senegalensis*, *Barbus barbus*) were included to root the tree. This tree (Figure 3) illustrates the separated position of clade 1, which is genetically very distinct from the other *G. barreimiae*, even tough the localities Wadi Bani Awf and Wadi Hat are geographically rather close to those represented in clade 3 (Figure 1a). The fact that *G. rufa *has smaller distances to each of the *G. barreimiae *clades than those found within *G. barreimiae *suggests that the species *G*. *barreimiae *as currently defined might not be monophyletic.

**Figure 4:**
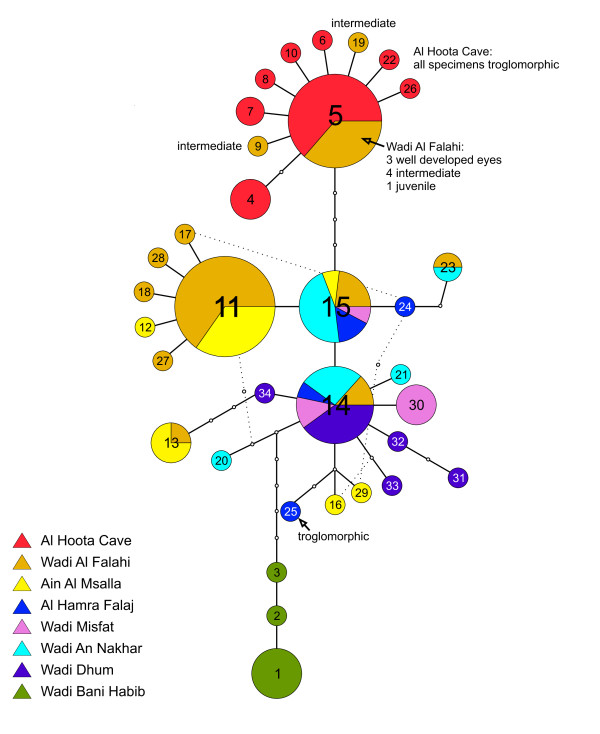
**Minimum spanning network of Clade 3 based on CR1 sequences**. Each dot represents a substitution; size of the circles corresponds to the number of individuals that share this haplotype. All specimens that did not have a surface phenotype are indicated.

A network of the complete *G. barreimiae *CR1 data set is shown in Additional file 1. The different clades and *G. rufa *are depicted in different colours. This network shows that the position of clade 1 with respect to the other *G. barreimiae *clades is quite distant. Clade 1 is found outside of *G. rufa*, this node is 31 substitutions away from the nearest node connecting *G. rufa *with clade 2. Clade 2 is separated by nine substitutions from clade 3 and clearly separated from *G. rufa *(28 substitutions). Details of clade 3 are shown in Figure 4.

### Phylogenetic relationships within clade 3

A TCS network based on CR1 sequences is shown in Figure 4. To enable a detailed display it includes only individuals from the populations comprising clade 3 (Wadi Dhum, Wadi An Nakhar, Wadi Misfat, Al Hamra Falaj, Al Hoota Cave, Wadi Al Falahi, Ain Al Msalla and Wadi Bani Habib). The individuals from the other five localities are genetically too distinct to be connected within the 95% connection limit (eight steps) and form separate groups, which are not shown. In the 122 individuals 34 different haplotypes were found. Four haplotypes (5, 11, 14, 15) are rather frequent occurring in more than 10 individuals, six haplotypes occur in two to six individuals, and the remaining 24 haplotypes occur only once. The most interesting finding is that all individuals from Al Hoota Cave are found within one haplogroup (5). It consists of one main haplotype, whereas the remaining haplotypes differ by one (eight haplotypes) or two (one haplotype) substitutions. This haplogroup not only contains the specimens from Al Hoota Cave but also includes 10 individuals from Wadi Al Falahi. The main haplotype of this group differs by four substitutions from another main haplotype that occurs in 13 individuals from five different localities. The network clearly shows that Wadi Bani Habib is the only locality that does not share any haplotype with individuals from other localities. Like Al Hoota Cave it is restricted to one haplogroup, while all other localities are genetically mixed with haplotypes shared between the different localities. The central haplotype 15 is separated by only one substitution from two other common haplotypes (11 and 14).

The haplotype diversities in Al Hoota Cave (0.67) and the adjacent localities Wadi Al Falahi (0.76) and Ain Al Msalla (0.68) are similar, while the nucleotide diversity in Al Hoota Cave (0.28) is much lower than in the other two localities (0.72 and 0.74). This is because the variation within Al Hoota Cave population consists mainly of single substitutions.

In a mismatch distribution analysis the Al Hoota Cave population showed a unimodal distribution indicating recent population expansion after a bottleneck [26]. In contrast the population consisting of the remaining localities from clade 3 showed a more ragged distribution generally fitting a distribution expected under a growth/decline model. In the Al Hoota cave population the Fu's *Fs *test statistic [27] and *R_2 _*statistic [28] were both significant (p < 0.01; p < 0.001) rejecting the hypothesis of constant population size. Clade 3 (except Al Hoota Cave) was significant for Fu's *F_s _*test statistic (p < 0.001), but not for the *R_2 _*statistic (p = 0.091). It has been shown that for small sample sizes *R_2 _*statistic performs better, whereas for larger sample sizes Fu's *F_s _*test statistic performs better [28].

### Troglomorphic state of the investigated individuals

Troglomorphic features are fully developed only in later stages. For juvenile fish (larvae) this prevents unambiguously evaluating, whether a particular individual would have developed to a troglomorphic phenotype or not. The sampled fishes were of different developmental stages. The largest individuals collected in the surface waters measured 67 mm SL (standard length), the largest from the Al Hoota cave were 47 mm in length. Our measurements confirm the finding of [14] that the troglomorphic phenotype is not yet well displayed in individuals smaller than 24 mm. Of the 72 individuals from the cave, only one specimen with 6 mm SL had well-developed eyes. Individuals between 14.5 and 23 mm (11 specimens) had partly reduced eyes. In the larger individuals of this group the eyes were sunken but still visible. All the other fishes that derived from Al Hoota Cave (>23 mm SL) had troglobiotic morphology. In six individuals of this group, faint remnants of eyes were visible, whereas the remaining specimens showed completely reduced eyes and no pigmentation. The distributions of haplotypes of troglomorphic and intermediate individuals are indicated in Figure 4.

**Figure 5:**
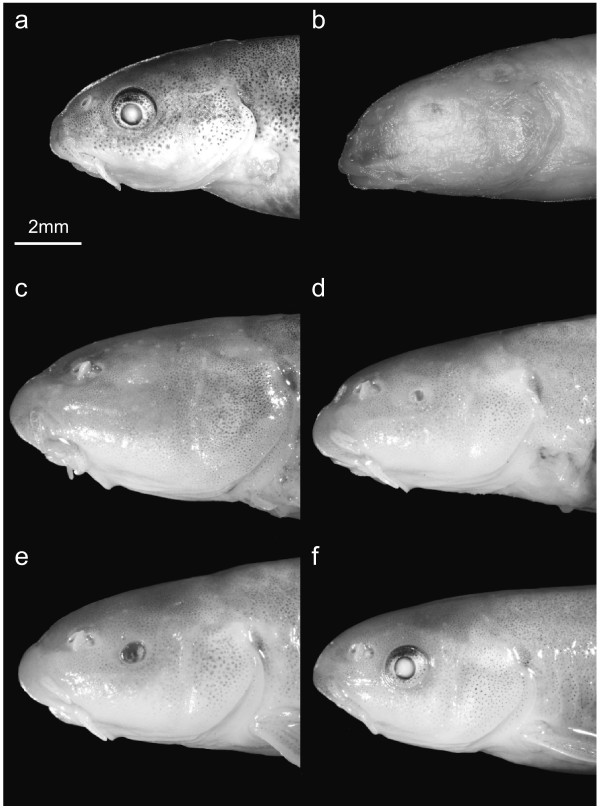
**Different phenotypes of *G. barreimiae***. a: surface fish from Wadi Al Falahi, b: troglomorphic fish from the Al Hoota Cave, c-f: different morphs of fish caught in the Wadi Al Falahi, which all belong to the "cave haplogroup". Troglomorphic state of individuals: b and c-e: clearly reduced eyes, a and f: eyes appear to be functional. Fish lengths (SL): 26 - 31 mm.

Troglomorphic characters were missing in nearly all individuals from surface populations. One exception was an adult individual collected from a different drainage system (Falaj), an irrigation channel near the town Al Hamra. This specimen was clearly troglomorphic (31 mm SL), suggesting that other troglomorphic populations might exist beside Al Hoota Cave. The haplotype (25) of this specimen is genetically only distantly related (2%) to the blind specimens from Al Hoota Cave. Other exceptions included some individuals collected in the ponds close to the cave exit in the Wadi Al Falahi on the 2^nd ^of December 2006 after a flood which brought big amounts of water from the cave to the surface. All 17 individuals showed a very weak pigmentation and the eyes were of variable size or not visible at all (Figure 5). Nine of them had clearly reduced eyes (27 - 45 mm SL), while eight had eyes that appeared to be functional (25 mm - 30 mm SL); we did not, however, perform any anatomical analysis. In addition, four individuals collected on the 24^th ^and the 26^th ^of January 2001 in Wadi al Falahi were also microphthalmic (49 - 55 mm SL). All other fish collected from surface waters (114; 25 of these below 14 mm SL) had well-developed eyes and full pigmentation.

## Discussion

### Genetic differentiation between troglomorphic fish of Al Hoota Cave and surface-dwelling fish of Wadi Al Falahi

What do the results reveal with respect to the origin of the troglomorphic forms? The main question of the study was whether the two morphotypes of *G. barreimiae *are genetically differentiated and whether gene flow occurs.

In the network (Figure 4) all individuals from Al Hoota Cave belong to one haplogroup ("cave haplogroup"), but this group contains also a few individuals from adjoining Wadi Al Falahi. Hence there is no strict correspondence between haplogroups and the two ecological environments. The "cave haplogroup" shows a pattern which suggests a founder effect caused by a single invasion into the cave followed by fast expansion; this is also supported by the mismatch distribution analyses. The presence of surface-dwelling individuals in this haplogroup might be explained by incomplete lineage sorting, which we consider unlikely for the following reasons: All Al Hoota specimens belong to one haplogroup. This "cave haplogroup" is not found in any other locality except in a few individuals collected in Wadi Al Falahi at the exit of Al Hoota Cave. Moreover, the vast majority of Wadi Al Falahi samples belong to several different lineages (i.e. haplotypes 13, 14, 23, 15, 11, 27, 18, 28, 17; Figure 4) and only a single haplogroup is shared with individuals in the cave. None of the other various haplotypes found in Wadi Al Falahi was detected in Al Hoota specimens. Finally, most of the individuals of Wadi Al Falahi possessing cave haplotypes show troglomorphic characters. In summary these facts indicate that the extant populations of Al Hoota and Wadi Al Falahi are not completely separated. Two potential scenarios explain how the populations might get into contact. (i) Although most of the water bodies in the wadi dry out frequently, fishes with eyes appear within a few days after rainfalls in the surface water (observation by HS and RS). Hence, fish might retreat into the groundwater, where they might get into contact with individuals of the cave population. For the surface dwelling fish, however, these environmental conditions might not be suitable for breeding. (ii) During floods, fishes might be sometimes washed out from the cave into Wadi Al Falahi. Up to 200 m^3^/sec [29] can flow out from the Al Hoota Cave into the Wadi Al Falahi after heavy rain. Concerning the direction of gene flow, our data support a sporadic translocation of individuals from Al Hoota Cave into the Wadi Al Falahi. From the ten individuals of the "cave haplogroup" caught in Wadi Al Falahi, six had reduced eyes. If gene flow was acting in both directions, some individuals with intermediate phenotypes or with "surface" Wadi Falahi haplotypes would be expected in Al Hoota Cave. Neither of these cases was detected and thus the most likely explanation is that occasional gene flow occurs from the cave to the surface, only. This could explain both the presence of samples with "cave haplotypes" and the occurrence of intermediate phenotypes in Wadi Falahi. The question, however, is whether the blind individuals can reproduce in surface water bodies. One would assume that blind fish have a low probability of surviving long and reproducing in surface populations because of impairment by radiation or by high predator pressure (e.g., from water insects, fish and birds). Gene flow is therefore expected to be quite low between surface- and cave-dwelling fish.

Besides the various intermediate forms, four individuals of the "cave haplogroup" from the Wadi Al Falahi possessed eyes that resembled the surface phenotype (Figure 5). It remains unknown whether the eyes of the latter individuals are functional: to date no systematic morphological investigation has been performed, whether there is a plastic response of eye development in the presence or absence of light. A single report by Banister [14] notes that the development of the optic lobes was influenced by light. Behavioural changes due to light exposure during development have been documented by [30]: light reared juvenile fish showed significantly higher photophilic behaviour than dark reared juvenile individuals. Should the eyes in the four above mentioned individuals be functional, then the most plausible explanation is that these individuals are hybrids between the two populations (or their descendants) that retained the cave haplotype but are able to develop functional eyes. In summary, our data indicates that the troglomorphic individuals of *G*. *barreimiae *living in Al Hoota Cave sporadically interbreed with individuals of the adjacent wadis.

Another question concerns the age of the cave population. An answer would provide indirect hints about the underlying mechanisms for the development of troglomorphic features. Surface and cave forms were crossbred in the laboratory (Wilkens, personal communication in [30]), suggesting that the two genomes are still compatible. If the eyeless condition was a genetic trait that became fixed due to relaxed selection pressure for maintenance of vision-related traits in the cave, then this would require some time. In this context the divergence time of lineages is of interest and dating of the split of the "cave haplogroup" based on genetic distances would be desirable. This is problematic because there is no reliable calibration for a molecular clock of *G. barreimiae*. The cave is of Plio-Pleistocene age, roughly one to three million years old. This might be set as the earliest date for the origin of the cave population. Nonetheless, the "cave" and the "surface" haplogroups may already have split some time before the intrusion into the cave. It is also possible that the cave population originated much later, and/or from a surface population with a different (more closely related) haplotype, which we either did not sample or which does no longer exist.

Despite these uncertainties, the observed genetic differentiation between sequences of the "cave haplogroup" and those from the surface populations is low (minimum 0.91% in the CR1 fragment). P distances are in the range of those found within several surface populations of *G. barreimiae*. Attempts have been made to calibrate a substitution rate for the *cytochrome b *(*cytb*) gene in cyprinids: 0.53% per million years [21] and 0.76% per million years [22]. For the mitochondrial CR no calibration exists, but it can be assumed that this section, due to less selective constraints, has a higher substitution rate compared to *cytb*. Applying the rates for the *cytb *gene for our CR1 data set would most probably result in an overestimate of the actual divergence time of epigean and hypogean *G. barreimiae*. Anyway, a rough calculation based on the distance of 0.91% and the two above mentioned rates yields a split below 1 mya (0.86 or 0.60 mya, respectively). This would disagree with the results of Colli et al. [23], who estimated the divergence time between epigean and hypogean *G. barreimiae *between 0.8 - 2.0 Mya based on a 795 bp sequence of *cytb*. This calculation remains dubious because it is unclear whether those sequences [23] represent the functional *cytb *gene or nuclear pseudogenes (numts - copies of mitochondrial genes that have been translocated into the nuclear genome) or chimeras of both. In the study of [31] the *cytb *sequences were composed of two independently generated PCR-products. Our preliminary experiments revealed the presence of *cytb *numts in this genus. We used primers that bind in the flanking t-RNA genes to amplify the whole gene (1140 bp) in one fragment (data not shown). Comparing the complete *cytb *sequences obtained in our study with those of [31] shows that in the 3'-section they are almost identical; while in the 5'-section they are quite different (8% p-distance). The most plausible explanation is that [31] presented a sequence chimera. An extensive investigation of *cytb *in this genus would be necessary to draw final conclusions and to disentangle authentic mtDNA sequences and numts. In any case, it is currently impossible to use *cytb *data for any attempt to date splits in *G. barreimiae*.

The very low genetic distances between cave and surface populations (Al Hoota/Wadi Al Falahi) suggests that the Al Hoota population is quite young. This indicates an evolutionary mechanism for troglomorphic characters that differs from the simple accumulation and fixation of neutral mutations. The results favour a selection-driven hypothesis as was suggested for *Astyanax*, where increased *shh *expression proved to cause eye degeneration and enhanced forebrain and taste bud development [5-8]. In the evolution of troglomorphic phenotypes, however, the same genes are not necessarily always have to be involved, and selective pressures influencing different cave species may not be the same all over the world. Factors affecting a cave population can vary widely (e.g., composition of the cave coenoses, availability of trophic resources, water regime, frequency of exchanges with the surface environment, etc.). Differences in physiology or the presence of preadaptations to cave life must also be considered, along with phenotypic plasticity and epigenetic effects. The effect of different environmental pressures can be particularly strong if the development of troglomorphic traits is due to epigenetic modifications or other changes in gene regulation. The plasticity of the depigmentation in *G. barreimiae*, which is reversible to some extent in the presence of light (observation by HS, [13]), is in accordance with the presumed recent origin of the cave population.

A very remarkable finding is that the single blind fish from Al Hamra Falaj surface locality had a haplotype (25) which is highly divergent from the Al Hoota Cave haplogroup. It did not occur in any other individual and is closely related to a haplogroup comprising individuals from various surface populations (Figure 4). We assume that this adult individual, which was caught in an irrigation channel, originally derived from a yet unknown cave population where the troglomorphic phenotype has developed independently. Convergent evolution of cave fish populations from different surface populations has also been documented for *Astyanax *species [17,18,32]. Alternatively, the phenotype of this specimen could be ascribed to a recent mutation with pleiotropic effects, and the individual by chance survived until maturity. The latter scenario, however, seems highly unlikely because, as far as it is known from other cave fish, aborted eye development is a multifactorial trait in which at least six genes are involved and, in addition, the lack of pigmentation is caused by different genes [5].

### Phylogeography and taxonomic considerations

Within clade 3 (Figures 3 and 5) the populations Wadi Al Falahi, Ain Al Msalla, Al Hamra Falaj, Wadi Misfat, Wadi An Nakhar and Wadi Dhum are genetically closely related and do not show any geographic structure. Within most of the localities, we found various haplotypes which are shared between different localities. This suggests frequent gene flow between localities. The wadis are usually not connected on the surface, but underground water bodies might exist which enable migration between the wadis. Flooding may also connect wadis. Another explanation would be that a former variable panmictic population was split only very recently through the separation of the wadis. The age of the youngest dripstone activity in Al Hoota Cave dates back 6,000 - 10,500 years [33]. This date corresponds with the last more humid period, in which the contact of different populations in permanent rivers within the same river system appears likely. Although water bodies are currently separated and thus gene flow is interrupted, lineage sorting in the current populations may not be completed. The geographically more distant locality Wadi Bani Habib (35 km away from the next population sampled) is genetically homogeneous, comprising three connected haplotypes not found elsewhere. This group is separated from the next main haplotypes by a minimum of 1.5% sequence divergence (six substitutions), suggesting longer isolation of this population.

The population of Wadi Bani Khalid (clade 2 Figure 3), besides Wadi Mansah, the only sample from Al Hajar Ash Sharqi (Western Hajar), is not only geographically the most distant one (200 km), it is also genetically rather distant (average p-distance to clade 3: 4%). The individuals from this population were described as a separate subspecies, *G. barreimiae gallagheri*, which is morphologically close to the nominate subspecies but differs in shorter fins and a higher number of dorsal fin rays [34]. Our results (i.e., the distinct mitochondrial lineage) are in accordance with this classification. Note, however, that many regions have not been sampled so far, for example we have no samples of the surrounding populations of Wadi Bani Khalid. It cannot be excluded that continuous geographic sampling would also reveal more continuous genetic relationships. The fact that neither this study nor any previous molecular study included fish from the type locality of *G. barreimiae *(Buraimi/Al Ain, which are nowadays densely populated) makes it difficult to address the intraspecific taxonomy of *G. barreimiae *based on the current state of knowledge.

The situation is different concerning the remarkably high genetic distances that were found between clade 1 (Wadi Hat, Wadi Bani Awf1, Wadi Bani Awf2 and Wadi Mansah) and the other clades. In fact, the genetic analyses question whether the individuals of clade 1 belong to *G. barreimiae *because the distances between this clade and the other lineages of *G*. *barreimiae *are in the same range as those between clade 1 and *G. rufa*. Our data do not resolve the phylogenetic relationships with a high statistical support. Although the specimens from these localities resemble *G. barreimiae *at first sight, no morphological analyses have been performed that definitely support this assignment. Thus, we cannot exclude the possibility that the populations of clade 1 represent a different species. Any taxonomic revision of *Garra *in Southeast Arabia should include phylogenetic analyses of samples from the topotypic locality or at least of a population close to the type locality. Nonetheless, the high genetic distances can be explained by the fact that these populations underwent long-term isolation because the water bodies they inhabit are separated by a watershed.

## Conclusions

Our data indicate that the troglomorphic cave population is of quite recent origin, supporting the hypothesis that selection drives the evolution of troglomorphic traits. In this context pleiotropic effects might play an important role as has been shown for *Astyanax*. There seems to be some gene flow from the cave population into the adjacent surface populations. Nuclear markers need to be employed to corroborate this result and to assess the amount of gene flow in more detail. The single blind individual found at a geographically distinct surface locality is genetically differentiated from Al Hoota Cave, indicating the probable existence of another cave population of *G. barreimiae*. The population at Wadi Bani Khalid is genetically separated, while the other surface populations from separated wadis seem to be sporadically in contact or have been in contact until recently. Certain populations belong to a very distant lineage. Based on the genetic marker used, the monophyly of *G. barreimiae *cannot be confirmed.

## Methods

### Sample collection and DNA extraction

From 2001 to 2008, *G. barreimiae *were sampled by H. Sattmann, R. Seemann, R. Illek and E. Fischer in the Western and Eastern Al Hajar Mountains in the Sultanate of Oman. A total of 208 *G. barreimiae *specimens were collected at 12 localities (Figure 1a and 1b). One of the populations was located within Al Hoota Cave (72 individuals, Figure 1b), while all the others were surface populations. Sample localities, abbreviations and numbers of specimens are listed in Table 1. Collected animals were preserved in 80% ethanol and are stored in the scientific collections of the NHMW. In addition, we included five specimens of *Garra rufa*; two of them were obtained (Gruf-1 and Gruf-2) from a breeding stock (originating from Kangal, Turkey; Paul Hofer, Wiener Neustadt, Austria; "rent a fish", 04/2006), while the others are specimens of the fish collection of the Museum of Natural History Vienna: Gruf-6 (Baykan, Siirt, Turkey, NMW 85794), Gruf-11 (Firat Nehri Birecik-Urfar, Turkey, NMW 87890), and Gruf-13 (Pella, Jordan, NMW 94980). From all specimens a tiny piece of the right pectoral fin was cut off for DNA extraction. All material collected between 2001 and 2006 was extracted by incubation of tissues in a 10% Chelex (Bio-Rad) solution containing proteinase K (0.5 mg/ml) for 4 h at 56°C (with agitation). Subsequently, extractions were heated to 95°C for 5 min and centrifuged for 1 min. The supernatant was purified using the QIAquick PCR Purification Kit (Qiagen) with a final volume of 30 - 70 μl elution buffer. DNA extraction from all samples collected in 2007 and 2008 was performed using the DNAeasy blood and tissue kit (Quiagen) following the manufacturer's protocol and using an elution volume of 80 μl.

**Table 1:** Sampling localities and their abbreviations (Abbr.)

Locality	**Abbr**.	col/gen	Phenotypes	Individual IDs
Al Hoota Cave	AH	72/25	troglomorphic	***Gbar8*, **Gbar9, Gbar10, Gbar11, **Gbar12**, Gbar20, ***Gbar21*, *Gbar22***, Gbar40, Gbar42, **Gbar43**, Gbar44, Gbar79, Gbar80, Gbar81, Gbar82, Gbar83, **Gbar84**, Gbar85, Gbar86, Gbar169, **Gbar170**, Gbar171, Gbar172, Gbar173
Wadi Al Falahi	WF	55/38	six intermediate otherwise surface	Gbar17, Gbar25, Gbar46, Gbar47, Gbar48, Gbar49, Gbar50, Gbar54, Gbar55, Gbar112, Gbar113, Gbar114, Gbar129, Gbar131, Gbar132, Gbar133, Gbar134, Gbar135, Gbar136, Gbar137, Gbar138, Gbar139, Gbar140, **Gbar141**, Gbar142, Gbar143, Gbar144, Gbar146, Gbar148, Gbar149, Gbar150, Gbar151, Gbar211, Gbar212, Gbar213, Gbar214, Gbar215, Gbar216
Ain Al Msalla	AM	21/16	surface	Gbar13, ***Gbar14***, Gbar15, ***Gbar16***, **Gbar31**, Gbar32, **Gbar33**, Gbar67, Gbar87, Gbar88, Gbar89, **Gbar90**, **Gbar91**, **Gbar92**, Gbar94, Gbar95
Wadi Bani Awf1	WA1	6/6	surface	Gbar60, Gbar61, Gbar62, **Gbar63**, Gbar64, Gbar65
Wadi Bani Awf2	WA2	4/3	surface	Gbar28, Gbar29, Gbar30
Wadi Bani Khalid	BK	5/5	surface	**Gbar57**, **Gbar58**, **Gbar59**, **Gbar66**, Gbar68
Wadi An Nahkhar	WN	13/13	surface	Gbar153, Gbar154, **Gbar155**, Gbar156, **Gbar157**, **Gbar158, Gbar159**, Gbar160, Gbar161, **Gbar162**, Gbar163, **Gbar174**, Gbar175
Wadi Bani Habib	BH	8/8	surface	Gbar164, Gbar165, Gbar166, Gbar167, **Gbar176**, Gbar177, **Gbar178**, Gbar179
Al Hamra Falaj	HF	5/5	all but one surface	Gbar180, Gbar181, **Gbar182**, **Gbar183**, Gbar184
Wadi Hat	HT	6/6	surface	Gbar185, **Gbar186**, **Gbar187**, Gbar188, Gbar189, Gbar190
Wadi Misfat	MI	7/7	surface	Gbar191, Gbar192, Gbar193, Gbar194, Gbar195, Gbar196, Gbar197
Wadi Mansah	WM	3/3	surface	Gbar208, Gbar209, Gbar210
Wadi Dhum	WD	10/10	surface	Gbar198, Gbar199, Gbar200, Gbar201, Gbar202, Gbar203, Gbar204, Gbar205, Gbar206, Gbar207

Number of individuals collected (col) at each locality and numbers of individuals analysed genetically (gen). Individuals from which a genetic sequence was obtained are listed (Individual IDs). Bold individual IDs indicate specimens from which CR1 and CR2 were analysed. Italic and bold individual IDs indicate specimens from which the complete CR was analysed. The phenotypes were analysed in individuals above 24 mm SL, because below this size there is no clear difference between troglomorphic and surface phenotype.

### Morphological evaluation

All individuals were evaluated concerning troglomorphic characters (eye development, pigmentation). When the iris and the pupil were completely visible the eyes were evaluated as functional. However, as all *G. barreimiae *(even those from the cave population) develop eyes in their early stages, the evaluation of whether they are troglomorphic or not is meaningful only in individuals above a certain size (>24 mm, [14]). We measured the standard length (from the tip of the snout to the root of the caudal fin, SL) of all specimens and evaluated only individuals above 24 mm. From selected individuals we took photographs (e.g., Figure 5) of the anterior part (from the tip including at least the whole operculum; 5.6 times magnification) and of the eye region including the nostrils (20 times magnification). Additional photographs are available from the authors on request.

### PCR amplification and sequence analysis

The following primers were used for PCR amplification: Gar-Thr1+ (5'-GCATCGGTCTTGTAATCCGA-3'), TDK_DGbar1- (5'-CCTGAAATAGGAACCAGATG-3'), CR1+_Gbar (5'-GCAGTAAGAGACCACCAAC-3'), 12SR5 (5'-GGCGGATACTTGCATGT-3'), CR2+_Gbar (5'-GAATCAAGGGCAATAATAGTGGG-3'), CR3-_Gbar (5'-TCTGGGTGGTTTTGTGTTGC-3'), CR4-_Gbar (5'-TTGGGCGTCGGCGGTGAGAG-3'). With the exception of primer 12SR5 (designed by Tomas Hrbek, pers. comm.) all primers were designed in the present study. From five individuals the whole mitochondrial control region (CR) was isolated by combining two overlapping fragments: 5' (Gar-Thr1+/TDK_DGbar1-); 3' (CR1+_Gbar/12SR5), the two external primers bind in the flanking *tRNA-Thr *gene and in the *12SrRNA *gene, respectively. As some samples had low DNA quality, we chose the 5'-section (approx. 440 bp) for the comprehensive analysis (CR1, 150 individuals). From selected individuals (altogether 33) representing the various clades, an additional 68 bp overlapping fragment (CR2, 545 bp), which covers the region 3' adjacent to CR1, was amplified with the primer pairs CR2+_Gbar/CR3-_Gbar or CR2+_Gbar/CR4-_Gbar. Primers CR3- and CR4- bind partly or completely within the region of the putative *tRNA-Pro*, thus this region was not present in the alignment used for the phylogenetic analyses. Combination of CR1+CR2 led to an alignment of 861 bp. This data set included also several outgroup species that possess a *tRNA-Pro *gene at the standard position, directly between the *tRNA-Thr *gene and the CR. Since the gene is missing at this position in *Garra*, we excluded this part from the CR1+CR2 alignment, which starts with position 21 of the CR of *G. barreimiae*.

PCR was performed with 35 reaction cycles on a Master gradient thermocycler (Eppendorf) in 25 μl with 0.5 U Dynazyme DNA polymerase (Finnzyme OY), 1 μM of each primer and 0.2 mM of each dNTP (Boehringer Mannheim). Control reactions of both DNA extraction and PCR amplification were performed. PCR products were purified using the QIAquick PCR Purification Kit (Quiagen) and sequenced directly, or they were extracted from agarose gels using the QIAquick Gel Extraction Kit (Qiagen) and cloned prior to sequencing (TOPO TA Cloning Kit, Invitrogen). Sequencing of both strands was performed by AGOWA (Germany), direct sequencing of PCR products was performed using the same primers as in the PCR.

### Genetic data analysis

Sequences were edited in BioEdit version 5.0.9 [35]. The alignment of sequences from the genus *Garra *was straightforward because there were very few insertions or deletions. Besides *G. rufa*, the following sequences from GenBank were used as outgroup: *Cyprinus carpio *(X61010), *Carassius gibelio *(GQ985484), *Labeo senegalensis *(AB238968), and *Barbus barbus *(AB238965). The alignment including sequences from other genera was performed with t-coffee [36] and adjusted manually (no regions of the alignment were deleted prior to the analyses). Average p-distances (pairwise exclusion of gaps) were calculated using *MEGA *version 4 [37], which was also used to calculate NJ trees [38]. Nodal support was evaluated with nonparametric bootstrapping based on 1000 replicates. BI analyses were performed using MrBayes 3.1.2 [39] applying the following models of sequence evolution for nucleotide sequences according to the Akaike information criterion (AIC) as implemented in the program MrModeltest v2.2 [40]: HKY+G (for the CR1 data set) and TrN+G (for the combined: CR1+CR2 data set). Runs were started with random trees and performed for 2 million generations each with four Markov chains and a sampling frequency of every 100th generation. Those trees generated prior to stationarity were discarded as burn-in and were not included in the calculation of the consensus trees. An unrooted parsimony haplotype network (95% probability level) of clade 3 was constructed by the method of [41] with the software TCS version 1.21 [42], treating gaps as 5th character state. A full median-joining network [43] of the complete *G. barreimiae *CR1 data set was constructed with network 4.6.0.0 (available at http://www.fluxus-engineering.com), putting equal weight on each site. The numbers of haplotypes and the haplotype diversities were calculated with ARLEQUIN 3.11 [44]. Past demographic fluctuations were assessed by calculating the mismatch distribution [26] as implemented in DnaSP v5 [45] to fit the observed data with models of population expansion. As a statistical test for population growth, we calculated the *R_2 _*statistic [28] and Fu's *F_s _*test statistic [27]. In both cases small values suggest recent demographic expansion. Significance (α = 0.05) of both the *R_2 _*statistic [28] and Fu's *F_s _*test statistic were evaluated by comparison to a distribution generated from 10,000 random simulations of the data in DnaSP v5.

Analyzing demographic fluctuations requires having a reasonable assumption about what a population actually is and to have an appropriate sampling. We chose Al Hoota Cave (25 specimens) and the group of localities found within clade 3 (except Al Hoota Cave; 97 individuals) as the only two populations for which a mismatch analysis is suitable. For the other genetically isolated clades, a mismatch analysis is not meaningful: The sample size of clade 2 is too small, and clade 3 is composed of two geographically disjunct populations (see results).

The sequences determined in this study are deposited at GenBank under the accession numbers: CR1: JF709013 - JF709129; CR1+CR2: JF709135 - JF709162; complete CR: JF709130 - JF709134.

## Authors' contributions

LK, EH and HS planned the research. LK carried out the molecular genetic and data analyses and drafted the manuscript. EH participated in the molecular genetic analyses and helped to draft the manuscript. RS contributed to the manuscript with his speleological and geographic expertise. HS initiated the study, coordinated the sampling and helped to draft the manuscript. All authors (excluding RS - see below) read and approved the final manuscript.

## Supplementary Material

Additional file 1 **Median-joining network of CR1 haplotypes of *G. barreimiae *and *G. rufa***. The different clades (as found in Figure 2) are depicted in different colours: dark grey: *G. rufa*; light grey: clade 1; turquoise: clade 2; white: clade 3. Small black circles represent median vectors. The size of each circle is proportional to each haplotype frequency. Numbers on branches denote connection steps >1. Click here for file
