# Supplementary figures and images for: Genetic differentiation between cave and surface-dwelling populations of Garra barreimiae (Cyprinidae) in Oman
Source: BMC Evol Biol. 2011 Jun 20;11:172. doi: 10.1186/1471-2148-11-172 (PMC3146880; doi:10.1186/1471-2148-11-172)

Clade 1

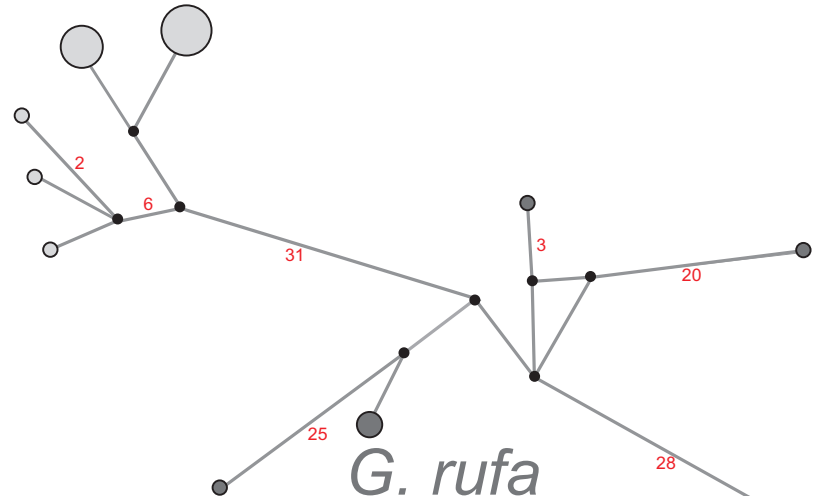

Clade 2

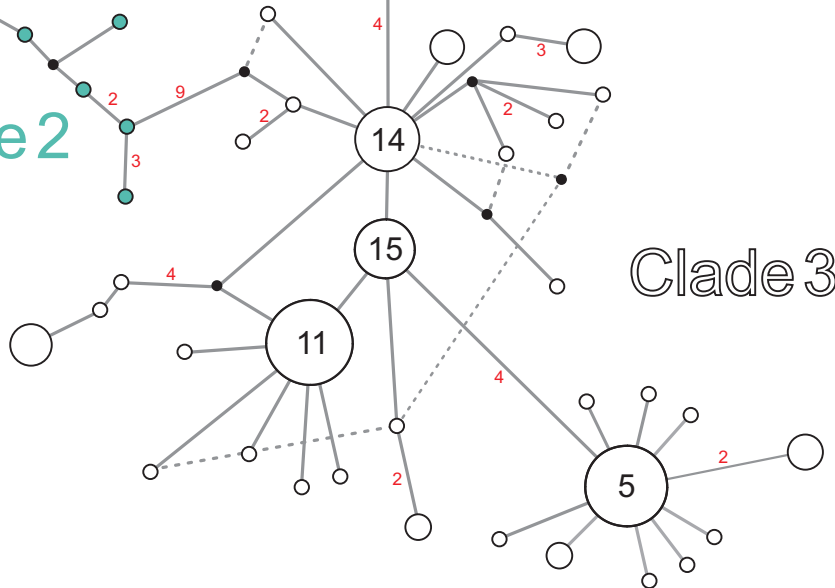

Supplement: Additional file 1 — Median-joining network of CR1 haplotypes of G. barreimiae and G. rufa. The different clades (as found in Figure 2) are depicted in different colours: dark grey: G. rufa; light grey: clade 1; turquoise: clade 2; white: clade 3. Small black circles represent median vectors. The size of each circle is proportional to each haplotype frequency. Numbers on branches denote connection steps >1. [file 1471-2148-11-172-S1.PDF]
